# Early Diagnosis and Hematopoietic Stem Cell Transplantation for IL10R Deficiency Leading to Very Early-Onset Inflammatory Bowel Disease Are Essential in Familial Cases

**DOI:** 10.1155/2016/5459029

**Published:** 2016-09-06

**Authors:** Neslihan Edeer Karaca, Guzide Aksu, Ezgi Ulusoy, Serap Aksoylar, Salih Gozmen, Ferah Genel, Sanem Akarcan, Nesrin Gulez, Tatjana Hirschmugl, Savas Kansoy, Kaan Boztug, Necil Kutukculer

**Affiliations:** ^1^Faculty of Medicine, Department of Pediatric Immunology, Ege University, Izmir, Turkey; ^2^Department of Pediatric Allergy and Immunology Department, Dr. Behcet Uz Children Training and Research Hospital, Izmir, Turkey; ^3^Research Center for Molecular Medicine of the Austrian Academy, Vienna, Austria

## Abstract

Alterations of immune homeostasis in the gut may result in development of inflammatory bowel disease. A five-month-old girl was referred for recurrent respiratory and genitourinary tract infections, sepsis in neonatal period, chronic diarrhea, perianal abscess, rectovaginal fistula, and hyperemic skin lesions. She was born to second-degree consanguineous, healthy parents. Her elder siblings were lost at 4 months of age due to sepsis and 1 year of age due to inflammatory bowel disease, respectively. Absolute neutrophil and lymphocyte counts, immunoglobulin levels, and lymphocyte subsets were normal ruling out severe congenital neutropenia and classic severe combined immunodeficiencies. Quantitative determination of oxidative burst was normal, excluding chronic granulomatous disease. Colonoscopy revealed granulation, ulceration, and pseudopolyps, compatible with colitis. Very early-onset colitis and perianal disease leading to fistula formation suggested probability of inherited deficiencies of IL-10 or IL-10 receptor. A mutation at position c.G477A in exon of the* IL10RB* gene, resulting in a stop codon at position p.W159X, was identified. The patient underwent myeloablative hematopoietic stem cell transplantation from full matched father at 11 months of age. Perianal lesions, chronic diarrhea, and recurrent infections resolved after transplantation. IL-10/IL-10R deficiencies must be considered in patients with early-onset enterocolitis.

## 1. Introduction

Inflammatory bowel diseases (IBD) are complex, mostly polygenic disorders characterized by relapsing intestinal inflammation. The incidence of IBD is growing among adults and children all over the world [1, 2]. Approximately 20% of patients are diagnosed before the age of 16 years [2–5]. Early-onset IBD (before 5 years) and very early-onset IBD (VEO-IBD, before 2 years) usually have severe and therapy-resistant courses, and the majority of VEO-IBD are caused by monogenic defects [2]. Multiple genetic defects in genes such as* IL-10*,* IL-10R*,* TTC7A*,* FOXP3*,* PLCG2*,* LRBA*,* G6PC3*,* IKBKG*, and* CYBB* are associated with IBD [3–5]. Interleukin-10 is an important anti-inflammatory cytokine in humans. Recently, deficiencies of interleukin-10 (IL-10) and IL-10 receptor (IL-10R), leading to defective STAT3 dimerization, have been shown to cause severe dysregulation of the immune system, resulting in VEO-IBD with perianal disease and the key to an effective therapy lies in early diagnosis and successful hematopoietic stem cell transplantation (HSCT) in these patients [2, 5].

Herein, a Turkish* IL10RB* deficient patient with a positive family history of early-onset enterocolitis cured with HSCT is presented.

## 2. Case Presentation

**Figure 1:**
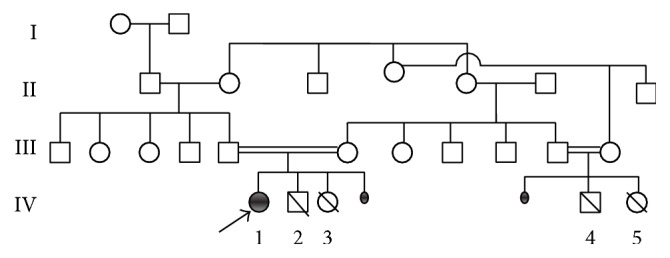
Pedigree of the consanguineous family. Patients' previous two siblings (IV-2 and IV-3) died at 4 months and 1 year of age due to sepsis and inflammatory bowel disease complicated with sepsis, respectively. She had two cousins who died at early infancy with unknown reasons (IV-4 and IV-5).

A 5-month-old girl was referred for recurrent infections of respiratory and genitourinary tract, chronic diarrhea, and hyperemic skin lesions. She was born at term with 3650 gr of birthweight, to second-degree consanguineous healthy parents. Her elder siblings were lost at 4 months of age due to sepsis and 1 year of age due to inflammatory bowel disease, respectively. She had two cousins dead at early infancy with unknown reasons. Pedigree is shown in Figure 1.

Her past medical history was loaded with frequent and severe infections. She was hospitalized at 10th day of life for sepsis, diarrhea (7-8 times a day), extensive skin rash on face and scalp, respiratory distress, fever, and urinary tract infection with* Klebsiella pneumoniae*. At 4 months of age, she had a gluteal abscess;* Klebsiella pneumoniae *was isolated from draining material. She was hospitalized at a public hospital for pneumonia and gastroenteritis at 5 months of age.

Her weight was 6.5 kg (10 percentile), height was 63 cm (3–10 percentile), and vital signs were normal at admission to our department at the age of 5 months. She had extensive erythematous skin lesions, coarse crackles on both chest areas, and anal fissure. Complete blood count was as follows: white blood cell (WBC) 20700/mm^3^ (normal WBC count for age: 6000–14000/mm^3^) (polymorphonuclear leukocytes 35.6%, lymphocytes 54.9%, monocytes 4.7%, and eosinophils 3.5%), hemoglobin 9.3 g/dL (normal: 10.5–14 g/dL), hematocrit 28.3% (normal: 32–42%), mean corpuscular volume 60.9 fL (normal: 72–88 fL), and platelet 577000/mm^3^ (normal: 150000–450000/mm^3^). Biochemical parameters were within normal ranges. C-reactive protein was 2.3 mg/dL (normal: <0.3 mg/dL), and erythrocyte sedimentation rate was 22 mm/hour (normal: <20 mm/hour). Urine analysis showed plenty of bacteria.* Extended spectrum beta lactamase positive E. coli* grew in the urine culture. High resolution computerized tomography revealed subsegmental atelectasis in right upper lobe apical and posterior segments. Abdominal ultrasonography was normal.

Primary immune deficiencies were first to be suspected in differential diagnosis because of her past clinical and familial medical history. Immunglobulins (IgG: 537 mg/dL, IgM: 52.6 mg/dL, IgA: 25.6 mg/dL, and IgE: 26.2 IU/mL) (age-related reference values; IgG 619 ± 208 mg/dL, IgM 78 ± 39 mg/dL, IgA 34 ± 25 mg/dL, and IgE <100 IU/mL) and complement levels (C3: 130 mg/dL, C4: 25 mg/dL) were normal for age. Percentages of lymphocyte subgroups were normal (CD3: 65.1% (normal: 51–79%), CD19: 18.6% (normal: 14–44%), CD3+CD4+: 47.7% (normal: 31–54%), CD3+CD8+: 13.9% (normal: 10–31%), and CD3-CD16+56+: 12.5% (normal: 5–23%)). Naive and memory T helper cell levels were normal for age. She did not have any elevation in TCR-gamma delta T cells. Transplacental maternal T cell engraftment was excluded by genetic analysis (PCR).* In vitro* T cell proliferation response to mitogens (PHA) was low. The quantitative determination of oxidative burst was normal, excluding chronic granulomatous disease. Foxp3 expression on CD4+CD25+ cells was normal. EBV and CMV-DNA were negative. Autoantibodies (anti-nuclear antibody, direct coombs test) were negative. Her immunoglobulin levels, although mostly normal initially, showed a fluctuating pattern with some lower values requiring intravenous immunoglobulin (IVIG) replacement regularly.

**Figure 2:**
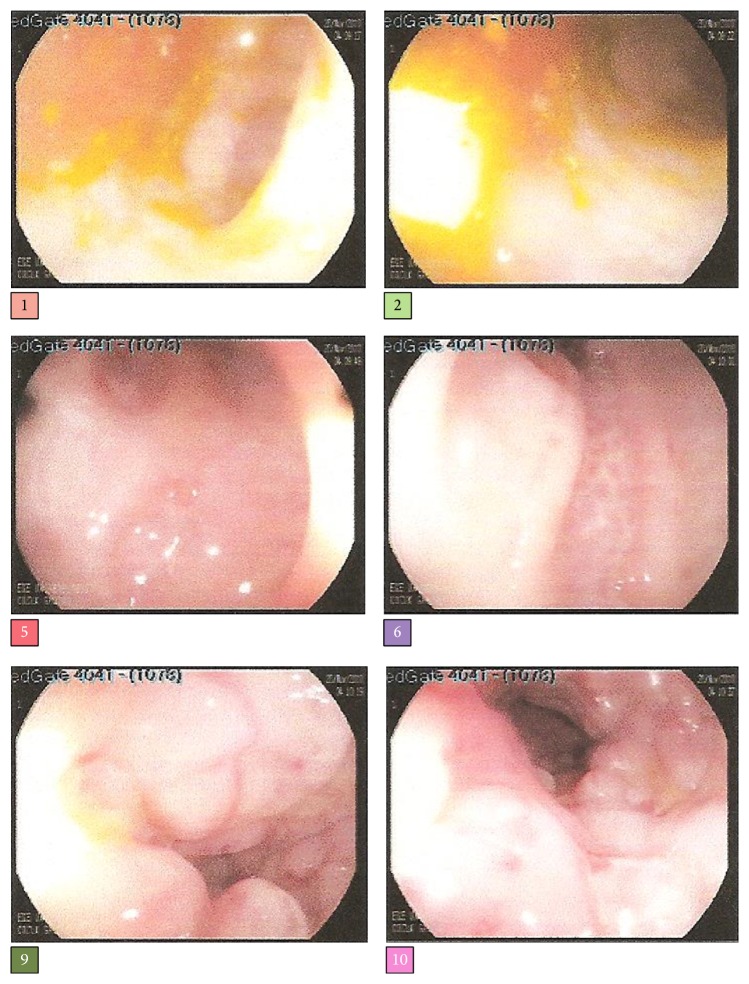
Colonoscopy images of the patient showing granularity of the right colon, exudative mucosal ulceration, pseudopolyps, laseration, and loss of haustration of the left colon, compatible with colitis.

She had ongoing diarrhea during follow-up. Stool investigations for bacteria, virus (rotavirus, adenovirus, and enterovirus), fungi, and parasites yielded negative results. Refractory diarrhea caused dehydration leading to central venous catheter replacement many times and several line infections with gram negative rods and* Candida* species, which prolonged hospitalization. The family noticed stool discharge through the vagina. Fistulography showed a rectovaginal fistula, which was treated by fistulectomy and anoplasty. Healing with scarred tissue led to colostomy decision. Although there was no blood in her stool, early-onset inflammatory bowel disease was considered because of refractory diarrhea, perianal disease, and positive family history. Colonoscopy revealed granularity of the right colon, exudative mucosal ulceration, pseudopolyps, laceration, and loss of haustration of the left colon, which was compatible with colitis (Figure 2). Histopathologic examination showed irregular architectural changes on the right colon and plasma cells in the lamina propria of the left colon. Macroscopic examination of the upper gastrointestinal tract by endoscopy was normal, while histopathology showed esophagitis, chronic active gastritis, and erosive bulbitis.

Recurrent infections, intermittent folliculitis, early onset colitis, and perianal disease leading to fistula formation and colostomy requirement together with positive family history in this patient suggested probable IL-10/IL-10R defects. A mutation at position c.G477A in exon of the* IL10RB* gene results in a stop codon at position p.W159X. This mutation has previously been published by Glocker et al. and STAT3 phosphorylation by IL-10 was shown to be abrogated [6].

**Figure 3:**
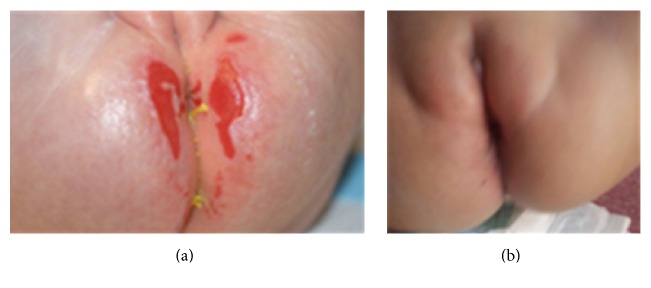
Perianal disease in the patient ((a) before HSCT, (b) after HSCT).

She underwent hematopoietic stem cell transplantation with 10/10 HLA (high resolution) matched related father at 11 months of age. She received myeloablative conditioning with the use of busulfan 4 mg/kg/day on days −5 to −2 and fludarabine 40 mg/m^2^/day on days −5 to −2, with rabbit ATG (Fresenius) 10 mg/kg/day on days −3 to −1. Gut decolonization was performed with the use of oral ciprofloxacin. Infection prophylaxis was intensified with acyclovir and amphotericin B (3 mg/kg/day). Enteral nutrition was given during the peritransplantation period. Cyclosporin A and short term methotrexate were used for graft versus host disease prophylaxis. Stem cell source was bone marrow. Counts of total nucleated and CD34+ cells were 4 × 10^8^/kg and 14 × 10^6^/kg, respectively. Neutrophil engraftment occurred at day +16. She was discharged at day +28. No severe complications were noted and perianal lesions, chronic diarrhea, and recurrent infections resolved (Figure 3). Tapering of cyclosporine was started at day +100 by 10% every week. Hematopoietic chimerism was determined using restriction fragment length polymorphism. She has full donor chimerism and is in excellent health condition after one year of HSCT.

## 3. Discussion

A number of primary immunodeficiency disorders such as selective IgA deficiency, common variable immunodeficiency, Bruton's disease, immune dysregulation-polyendocrinopathy- enteropathy X-linked (IPEX) syndrome, Wiskott-Aldrich syndrome, chronic granulomatous disease, NEMO deficiency, and combined immunodeficiency disorders are associated with gastrointestinal manifestations, namely, chronic diarrhea, giardiasis, nodular lymphoid hyperplasia, malabsorption, and inflammatory bowel diseases [4]. Mutations in* interleukin-10 receptor (IL-10R)* and* IL-10* genes have recently been discovered as an etiology for severe VEO-IBD [7, 8].

IL-10 is an anti-inflammatory cytokine produced by wide variety of hematopoietic cells such as regulatory T cells, macrophages, granulocytes, and dendritic cells [9, 10]. IL-10 has a critical role in maintaining the balance of the immune system by limiting the secretion of proinflammatory cytokines such as TNF-*α*, IL-1, and IL-6 and inhibiting the secretion of T helper 1 cytokines (IL-2 and IFN-*γ*). IL-10 provides protection against excessive immune responses and tissue damage cytokines [9]. Mice studies of IL-10 or IL-10 receptor (IL-10R) deficiencies have shown that IL-10 is a key cytokine for immune homeostasis of intestinal microbiota [7, 9].

Glocker et al. [6] performed genetic-linkage analysis and candidate-gene sequencing to nine patients with early-onset colitis and identified loss-of-function mutations in genes* IL10RA *and* IL10RB*, encoding the IL10R1 and IL10R2 proteins in 4 patients. Our patient had the same homozygous point mutation in exon 4 of* IL10RB* gene in 2 affected Turkish siblings (c.G477A, p.Trp159X). The authors showed that these mutations abrogate IL-10-induced STAT3 signaling by functional studies. The defects resulted in deficient STAT3 phosphorylation on stimulation with IL-10 and augmented efflux of tumor necrosis alpha and other proinflammatory cytokines, suggesting the lack of IL-10-dependent negative feedback on the homeostasis of the intestinal immune system [6].

Patients with IL-10/IL-10R defects usually present with early-onset enterocolitis and severe perianal diseases such as fissures, abscesses, or fistulas and may show other clinical features including chronic folliculitis, recurrent respiratory diseases, and arthritis. Abscess formation, anal fissures, and enterocutaneous or rectovaginal fistulas can complicate the disease and they frequently require surgical intervention such as our patient [6, 10–13]. Our patient had recurrent respiratory diseases and gastroenteritis with chronic folliculitis and was complicated two times with anal abscess and a rectovaginal fistula that needed fistulectomies. Protective colostomy was performed because of impaired wound healing. Additionally, IL-10/IL-10R deficiency patients have the risk of developing B-cell lymphoma [14–16]. Neven et al. [16] reported the frequency of EBV-negative-B-cell lymphoma as 36% (5 of 14 patients with a deficiency in the IL-10 pathway) at the age of 7 years.

Most of the patients defined in the literature were born to consanguineous parents [6, 15, 16]. Our patient was also born to first-degree cousin parents who had two children who died before 1 year of age, one with septicemia and the other with severe colitis.

Evaluation of the immune system reveled normal T and B-cells in number. The neutrophil function evaluated with oxidative burst test was also normal. She had mild hypogammaglobulinemia.* In vitro* T cell proliferation response to mitogens (PHA) was low. Engelhardt et al. [15] also reported the results of T cell proliferative assays in 3 patients, who all had reduced response to mitogens.

With all these clues, sequence analyses were performed and a homozygous stop-gain mutation in exon 4 of* IL10RB* gene confirmed the diagnosis of IL-10R deficiency. A homozygous mutation in intron 3 of the* IL10RA* (c.368-10C>G) was also identified in 3 related children with VEO-IBD and the authors emphasized the need for genetic diagnosis of mutations in conserved noncoding sequences of candidate genes [17].

IL-10 and IL-10R deficient patients are usually unresponsive to immunosuppressive therapies with corticosteroids, methotrexate, thalidomide, and even infliximab. Allogeneic HSCT is the current curative therapy [3, 5, 15]. Few patients with IL10 deficiency are reported to have allogeneic HSCT in the literature [5, 13, 15, 18]. Some of these patients received reduced intensity conditioning (RIC) which may be an option in case of poor clinical condition [5, 13, 18]. Beier et al. [18] performed RIC HSCT to 4 patients. Graft rejection was reported in one of them who received second successful HSCT. Engelhardt et al. [15] presented the clinical outcome in IL-10 and IL-10R deficient patients with or without HSCT. Colitis resolved in 3 of 9 patients who underwent HSCT. Two of the 6 patients without HSCT developed EBV-lymphoma, one died due to septicemia, and the remaining 5 patients had ongoing colitis, refractory to immunosuppressive therapy. Our patient did not have any further anal abscesses and fistulas after HSCT at 11 months of age; cutaneous folliculitis, chronic gastroenteritis, and recurrent respiratory tract infections resolved.

In conclusion, this case highlights the importance of early diagnosis of IL-10/IL-10R deficiency in patients with early onset enterocolitis, especially with severe perianal diseases and other clinical features including chronic folliculitis and recurrent respiratory diseases. Importance of family history that led to diagnosis and curative treatment for this case should also be emphasized. Cases with affected relatives should be evaluated carefully in terms of inherited diseases. Early HSCT has high positive impact on morbidity and mortality in VEO-IBD patients.
